# Periodically taken photographs reveal the effect of pollinator insects on seed set in lotus flowers

**DOI:** 10.1038/s41598-022-15090-0

**Published:** 2022-07-11

**Authors:** Mihoko Nagai, Yohei Higuchi, Yusei Ishikawa, Wei Guo, Tokihiro Fukatsu, Yuki G. Baba, Mayura B. Takada

**Affiliations:** 1 Faculty of Science and Engineering, Chuo University, 1-13-27, Kasuga, Bunkyo-ku, Tokyo, 112-8551 Japan; 2 Graduate School of Agricultural and Life Sciences, The University of Tokyo, 1-1-1, Yayoi, Bunkyo-ku, Tokyo, 113-8657 Japan; 3 Institute for Sustainable Agro-Ecosystem Services, Graduate School of Agricultural and Life Sciences, The University of Tokyo, 1-1-1, Midori-cho, Nishitokyo-shi, Tokyo, 188-0002 Japan; 4 Institute of Agricultural Machinery, NARO, 3-1-3, Kannondai, Tsukuba-shi, Ibaraki 305-8604 Japan; 5 Institute for Agro-Environmental Sciences, NARO, 3-1-3, Kannondai, Tsukuba-shi, Ibaraki 305-8604 Japan

**Keywords:** Ecological networks, Pollination

## Abstract

Understanding of pollination systems is an important topic for evolutionary ecology, food production, and biodiversity conservation. However, it is difficult to grasp the whole picture of an individual system, because the activity of pollinators fluctuates depending on the flowering period and time of day. In order to reveal effective pollinator taxa and timing of visitation to the reproductive success of plants under the complex biological interactions and fluctuating abiotic factors, we developed an automatic system to take photographs at 5-s intervals to get near-complete flower visitation by pollinators during the entire flowering period of selected flowers of *Nelumbo nucifera* and track the reproductive success of the same flowers until fruiting. Bee visits during the early morning hours of 05:00–07:59 on the second day of flowering under optimal temperatures with no rainfall or strong winds contributed strongly to seed set, with possible indirect negative effects by predators of the pollinators. Our results indicate the availability of periodic and consecutive photography system in clarifying the plant-pollinator interaction and its consequence to reproductive success of the plant. Further development is required to build a monitoring system to collect higher-resolution time-lapse images and automatically identify visiting insect species in the natural environment.

## Introduction

Understanding the complex interactions and mechanisms in pollination systems, especially those involving the predominant insect pollinators, has been a major focus of evolutionary ecological research^1–4^, agricultural production^5^ and conservation of endangered plants and insects^6,7^. For an accurate understanding of individual pollination systems, it is essential to identify effective pollinator species or taxa that contribute to the reproductive success of a focal plant species. Although many insects visit flowers, some may have no effect, or even negative effects, on plant fitness, such as through nectar robbing ^8^, pollen theft ^9^ or florivory^10^, so only limited visitors can be effective pollinators^11,12^. Moreover, the activity patterns of effective pollinators may not be constant all the time; they may vary with the flowering season^13,14^ and even over the course of the day, depending on external factors such as weather conditions^15,16^, the quantity and quality of rewards from the target plant^17^, constraints imposed by the insect’s own internal clock rhythm^18^, and biotic interactions with competitors^19^ or predators^20^. To get the full picture, we need to track the visitation pattern and its changes over time throughout the day and throughout the flowering period^21–23^ rather than make short-term observations, specially when the life-span of the flower and species allows it.

Most pollination studies have been conducted by direct visual observation in the field^24–27^, but they are time consuming and labour intensive. It is very difficult to continue direct observation from dawn to dusk without a break, or to record the number and individual species of flower visitors with the precise time of visitation if several visitors rush to a flower. In recent years, insect monitoring systems with automatic still or video cameras have been used in the field, and techniques for observing pollinators recorded by these devices have been developed^28–30^. Photographs and videos can keep recording for longer time without getting tired and can be reviewed repeatedly in order to record precise data of each flower visitation event. Successive observations using these devices have helped identify effective pollinator taxa^31–33^ or changes in pollinators’ activity with the time of day^21,22,34,35^. By using several cameras, they can also observe the patterns of pollinator visitation among several plants simultaneously^36^. But the most important point is to confirm whether the visitation by the pollinator is reliably linked to the reproductive success of the same flower. Very few studies to date have linked the observation of pollinator visitation throughout the flowering period to the reproductive success of the flower itself, under the complex biological interactions between species of multiple trophic levels.

One of the reasons for the lack of such studies is the difficulty in transporting and maintaining heavy and expensive equipment in harsh natural environments such as high mountains, rainforests or riversides. Although trail cameras have become popular in ecological and behavioural studies of wild animals^35^, the establishment of techniques and research methods for continuous recording of small, fast-moving insects is still in its infancy^37^. Therefore, it would be useful to establish photographic techniques and observation methods by using cultivated plants under controlled conditions. If technological developments can lead to smaller and cheaper equipment in pollination research, it might be possible to apply photographic systems and research designs to a wider range of taxa and environments^29^.

Here, we developed a system that can observe near-complete flower-visitations during the entire flowering period of a single flower of the insect-pollinated *Nelumbo nucifera* Gaertn. and track the reproductive success of the flower until fruiting. *Nelumbo nucifera* is a basal eudicot that diverged from the basal angiosperms in the Cretaceous period^38^. The explosive diversification of angiosperms during this period is thought to have been brought about by co-evolution with pollinators, including insects. As the flowers of *N. nucifera* close in the middle of the first and second days of flowering and do not reopen until early mornind^39^, we can get a near complete series of images of flower-visiting insects without the need to take photos in the dark. It also has the advantage of being under controlled cultivation, so that negative effects of resource limitation on fruit set^40^ can be eliminated and the independent effect of pollination can be isolated. In addition, the wide variation in floral traits among cultivars is suitable for analysing the effect of floral morphology on insect attraction. As medicinal components in lotus seeds have received much attention in recent years^41,42^, improving the efficiency of seed production can be an important horticultural mission for this species. The goals of this study were: (1) to compare the differences in flower visitation patterns among different insect groups, (2) to identify the meteorological factors that affect visitation patterns, (3) to identify the insect groups and the timing of visitations that contribute the most to seed set, and (4) to analyse the interaction between pollinators and their predators on the flower and the consequence for the flower’s reproductive success.

## Materials and methods

### Plant materials and study site

Sacred lotus (*Nelumbo nucifera* Gaertn.) is an aquatic perennial in the Nelumbonaceae, native to India. It is widely grown in Asian countries for ornamental, food and medicinal uses^43^. Many cultivars have been produced, with a wide variation in flower colour, petal type (single or double), flower size and other characteristics. The species has diurnal flowers; on the first and second days of flowering (Day 1 and Day 2), the flowers open early in the morning and close before noon, though on Day 3 and later, the petals close loosely, so the receptacles remain visible even at night^39,44^. Stigmas, which are arranged on the surface of the receptacle, become receptive on Day 1, but anthers dehisce on Day 2 (i.e., the flowers show temporal protogyny). The nectarless, thermogenic flowers^45^ are self-compatible but exhibit a small amount of automatic self-pollination^39,46^, so pollinators are needed for sufficient seed set. Beetles and bees are the main pollinators, along with flies in some Chinese populations^39^.

Field observations were conducted during the summer of 2018 at the Lotus Specimen Garden of the Institute for Sustainable Agro-Ecosystem Services, Graduate School of Agricultural and Life Sciences, The University of Tokyo, Japan (35°44′03″N, 139°32′22″E). More than 300 cultivars are grown and maintained in the Garden, including some derived from the related American lotus *Nelumbo lutea* Willd. Around 100 cultivars are grown in concrete ponds (2 m × 2 m square), and > 200 are grown in plastic containers (65 L). We got formal permission from the Institute to conduct the lotus experiments and collect the fruits prior to the study, and relevant institutional, national, and international guidelines and legislation followed for the present study. The individuals used in this study are maintained under the cultivation in the Garden.

### Experimental design and photography method

Twelve ornamental lotus cultivars with varied flower size, petal type and numbers of stamens (10–300) and pistils (4–32) were used (Supplementary Table S1). One of the authors, a manager of lotus cultivation in the Garden, identified the cultivars. Flowering time varied from late June to late August. One bud per cultivar was selected and marked, then photographic equipment (TLC200 Pro, Brinno, Taipei City, Taiwan) held inside a weather-resistant housing was fixed on a tripod and set in front of the bud (Supplementary Fig. S1). This equipment was provided with a time-lapse shooting function as standard feature. Photographs were taken every 5 s at 1280 * 720 resolution from 04:00 to 19:30 or 20:00, depending on day length, over the 3 to 4 days of flowering until petals and stamens wilted and dropped off. There was no need to replace the batteries (4 AA batteries) or storage (32-GB SD cards) during the course of a recording. Ripe fruits were harvested about 2 months after flowering, and those with mature seeds and seedless fruits were counted. Fruits that did not develop fully were cracked open to check for the presence of viable embryos. Fruit receptacles of two cultivars were unfortunately lost owing to bad weather, so the rest 10 cultivars were used for seed set analyses.

### Arthropod observations and identification by photograph

To acquire species references for identification of arthropods by photograph, we collected arthropods that visited other lotus flowers and identified them under a stereomicroscope. Using the reference specimens, 452,000 photos in total were reviewed by several researchers on a computer screen, and the number and taxonomic group of arthropods > 4 mm in length in contact with the upper surface of the receptacles or stamens were recorded (but not those perched on the petals or in flight). Arthropods under 4 mm in length (almost all observed were thrips) were excluded from the analyses because they were not likely to contribute to seed set in lotus flowers^39^ and could not be counted accurately. If two or more arthropods in the same taxonomic group were found in a photo, the number was summed. Individual arthropods were identified to the lowest taxonomic level that could be identified at the definition of this camera system: moths to order, bees in the Halictidae and spiders to family, honeybees to genus and others to species. Individuals that were only partly visible and difficult to identify even in sequential photos were classified as ‘unknown’. The number of times wasps and spiders, which may predate on pollinator insects, were photographed on any part of the surface of flowers was counted.

From direct observations of flower visits and the photos, we saw that the duration of stay during a single flower visit (visitation time) varied greatly among arthropod groups, so we calculated the visitation time for each group as follows. The visitation time *t* of an arthropod individual found in only one photo was always 0 < *t* < 10 s, but here we defined it as 5 s for approximation, and we calculated the visitation time of an arthropod taken in *N* consecutive photos as 5* N* s. We reviewed 12–34 randomly selected photo sets (the number varied depending on the number and variability of the photos of each group taken) of each arthropod group that we presumed showed the same individual continuously, and we estimated the mean visitation time of a single visit, $\overline{t}$. The approximate number of the visits during the whole flowering period, *v*, can be estimated as:

$$\begin{gathered} v = {\text{ total visitation time}}/{\text{mean visitation time}} \hfill \\ \,\,\,\, = \, \left( {{5 } \times {\text{ number of photos taken during total flowering period}}} \right)/\overline{t} \hfill \\ \end{gathered}$$

The weather conditions were determined by viewing the photos for evidence of rainfall and strong wind. Evidence was recorded as 1 (yes) or 0 (no) in each flowering hour. Strong wind was defined as shaking the flower by more than the width of a flatly opened flower (20–30 cm, depending on the cultivar). Temperature data were taken from the Nerima Meteorological Observatory (35°44.1′N, 139°40.1′E, ~ 5 km from the study site), which was the closest to the study site (http://www.data.jma.go.jp/obd/stats/etrn/select/prefecture00.php).

### Relationship between behavioural patterns of predators and prey on flowers

The behaviour of wasps visiting lotus flowers in the photos was classified as trying to hunt (‘hunting’), walking around to search for prey (‘searching’), standing still on the receptacle (‘waiting’; or maybe feeding on stigmatic secretions^39^), flying just above the flower with legs slightly on the petal (‘flying’), or resting on the petals (‘perching’). For hunting wasps, the target insects and the success or failure of the hunt were also judged from the photos before and after the hunt. The total numbers of each pollinator group just at the time of a wasp’s arrival, and 5, 10, and 15 s before and 5, 10, 15, and 300 s after the arrival, were counted, and the differences in change over time among the groups were compared.

### Statistical analyses

To test the hypothesis that weather and flower conditions affected flower visitation, a GLMM test was conducted for each insect group except for moths (very few of which were photographed), with the number of photos per hour as the response variable; the day of flowering, rainfall, strong wind, air temperature (°C), and the square of air temperature as explanatory variables; and cultivar as a random factor. The response variable was assumed to follow a Poisson distribution. For the analysis of wasps, the number of times each group of prey insects (bees, flies and beetles) was photographed was added to the explanatory variables.

To clarify how insect visitation contributes to the seed set of each flower, we conducted a three-step analysis. First, to determine the most effective day of flowering for seed set, we conducted a logistic regression analysis in which the number of photos of all flower-visiting pollinator insects on each day was used as the explanatory variable; the number of mature seeds (the response variable) was assumed to follow a binomial distribution, with the number of ovules in a flower as the upper limit. Before the analysis, we confirmed that there was no multicollinearity among the explanatory variables. Second, to determine the most effective pollinator group for seed set, we conducted a similar analysis by using the number of photos of each insect group taken on the day when the strongest relationship was found in the first analysis as an explanatory variable. For the insect groups and the day that were considered to be the most effective for seed set, we confirmed the change in Akaike’s information criterion (AIC) in logistic regression models of the number of photos taken during every 3-h time period as an explanatory variable when the time was shifted by 1 h^47^, and we identified the time period that had the strongest relationship with seed set. Finally, we conducted a logistic regression analysis in which the number of photos taken of the most effective pollinator group during the time period when the AIC value was lowest, petal type (single or double), number of ovules (an index of flower size) and Julian date (number of days from 1 April 2018 to day 1) were used as explanatory variables; the number of mature seeds per flower was used as the response variable. The response variable was assumed to follow a binomial distribution with the number of ovules as the upper limit. All analyses were conducted in R v. 4.0.3 software^48^.

## Results

### Flowering pattern of lotus

The petals of the 12 observed flowers began to open at about 04:30, just after sunrise. By about 05:00, corollas had opened to the extent that small flies and bees could burrow into the void (~ 2 mm diameter); this stage was defined as ‘flower opening’ (Supplementary Fig. S2a). On Day 1, the petals did not open completely (Supplementary Fig. S2b) and began to close 2–4 h after opening. By noon, the corolla was completely closed. (The stage when the corolla opening had decreased to that of ‘flower opening’ was defined as ‘flower closure’.) On Day 2, the flowering schedule was similar to that on Day 1, but the corolla opened almost fully (Supplementary Fig. S2c). On Day 3, the behaviours of the flowers were variable: the petals wilted by about noon (4 cultivars), the corolla closed at night (2 cultivars), or the corolla did not close, leaving the receptacles exposed (6 cultivars). Stigma surfaces became discoloured and unreceptive during Day 3 or 4 (Supplementary Fig. S2d). Some flowers retained petals on Day 5, but there was little pollen left in the anthers, so the anthers were considered not to contribute to pollination. The median durations when some receptacles and stamens were visible in the photos were 71 min (range, 0–247 min) on Day 1, 319 min (230–785 min) on Day 2, 482 min (217–875 min) on Day 3, and 556 min (0–860 min) on Day 4. The flower opening time was delayed by several hours in rainy weather, and in some cases the petals did not open fully in strong winds.

### Observed arthropods and their visitation patterns

**Figure 1:**
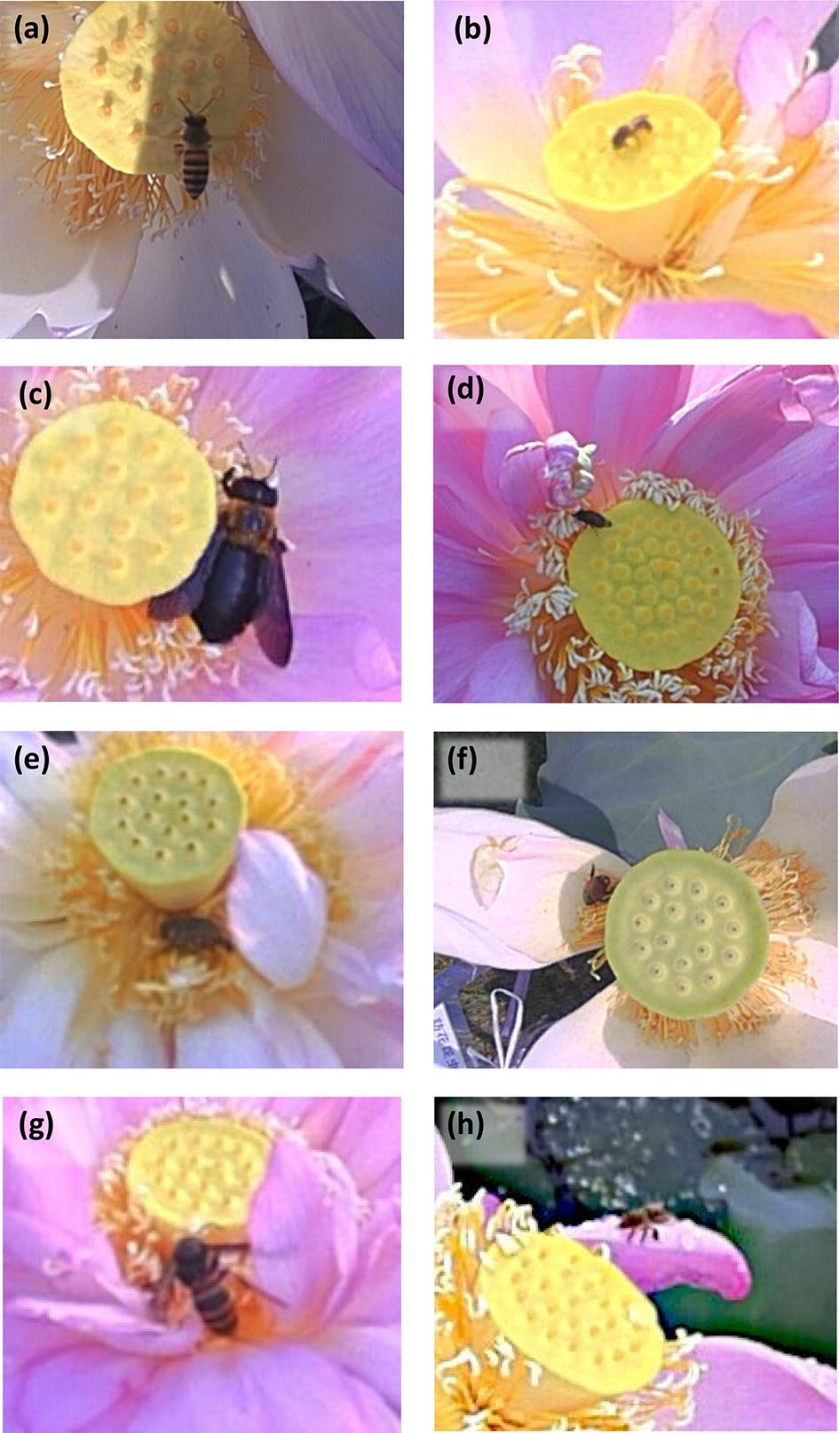
Examples of photos of arthropods visiting lotus flowers taken in the field of this study: (**a**) honeybee, (**b**) Halictidae sp., (**c**) *Xylocopa appendiculata circumvolans*, (**d**) *Stomorhina obsoleta*, (**e**) *Gametis jucunda*, (**f**) *Popillia japonica*, (**g**) *Vespa analis insularis*, and (**h**) spider.

Of the photos taken during the flowering period of the 12 flowers, 10,848 showed contact by insects in the Hymenoptera, Diptera, Coleoptera or Lepidoptera with the upper surface of the receptacles or stamens, which may contribute to pollination. We identified 12,831 individuals. In the Hymenoptera, honeybees (not distinguished between *Apis mellifera* and *Apis cerana japonica*), *Xylocopa appendiculata circumvolans* and bees in the Halictidae were found. In the Diptera, *Stomorhina obsoleta*, *Phytomia zonata* and other unidentified flies were found. In the Coleoptera, *Gametis jucunda*, *Popillia japonica* and other unidentified beetles were found. In the Lepidoptera, unidentified small moths were found. The main visiting species are shown in Fig. 1. The total numbers of photos taken in each taxon, including predators (wasps and spiders), are shown in Supplementary Table S2.

**Figure 2:**
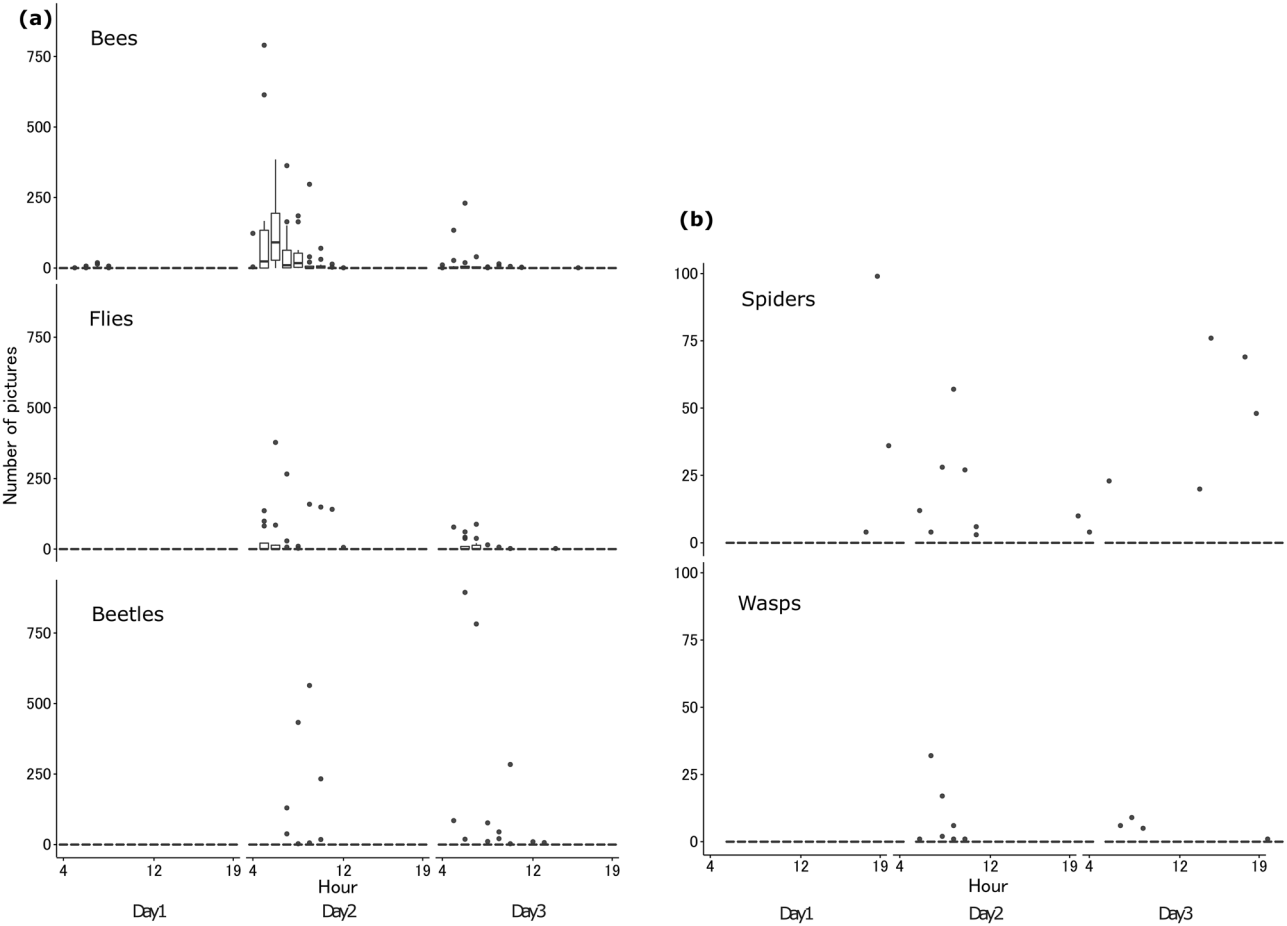
Time course of average number of photos of visitors to 12 lotus flowers, by flowering date and taxonomic group up to Day 3: (**a**) potential pollinators (bees, flies and beetles), (**b**) possible predators of pollinators (spiders and wasps).

As it was impossible to identify all arthropods to the species level, we categorized the flower visitors into four taxonomic groups of pollinators (bees, flies, beetles and moths) and two of predators (wasps and spiders) for subsequent analyses. The time course of the number of photos of each group (except for moths) per hour up to Day 3 is shown in Fig. 2. Bees (especially *Apis* spp.) were the most frequently observed insects throughout the flowering period, accounting for 45.3% of all flower visits by potential pollinators. Flower visits by bees were concentrated in the morning of Day 2 (Fig. 2a). Among them, honeybees had a short mean visitation time of $\overline{t}$ = 10.44 ± 8.01 s (± SD) (*n* = 34), and the estimated number of visits to the 12 flowers throughout the flowering period was 2,867. Halictidae bees were estimated to have visited flowers about 90 times, with $\overline{t}$ = 104 ± 218 s (*n* = 23). Fly visitation was also highest on Day 2, but it was still high on Days 3 and 4 (Supplementary Table S2). *Stomorhina obsoleta*, the most frequently photographed fly, spent about 5 min in a single visit ($\overline{t}$ = 278.0 ± 270.1, *n* = 23) and visited an estimated 41 times. Beetle visitation peaked on Day 3 (Supplementary Table S2), although it varied among the flowers (Fig. 2a). Two major species of beetles—*G. jucunda* ($\overline{t}$ = 591.8 ± 1376, *n* = 20; 11 visits) and *P. japonica* ($\overline{t}$ = 1344 ± 1,440, *n* = 12; 7 visits)—sometimes stayed, foraging pollen, for more than an hour in one flower.

Weather factors strongly influenced the number of flower-visiting pollinator insects recorded. The number of bees visiting flowers was significantly higher on Days 2 and 3 than on Day 1, and both rainfall and strong wind had significant negative effects (Table 1). Flies and beetles did not visit flowers at all during rainfall, but there was no clear difference among the Days (Table 1). For all insects, temperature had a significant positive effect and its square had a significant negative effect, suggesting an optimal temperature range for flower visitation, above and below which the visitation frequency decreased.

**Table 1:** Results of GLMM analysis examining the effects of weather factors and day of flowering on the number of photos featuring bees, flies, beetles and wasps visiting lotus flowers. The number of photos of each insect group per hour was assumed to follow a Poisson distribution. As weather factors, rainfall, strong wind, air temperature (°C), and the square of air temperature were analysed. For the analysis of wasps, the number of times each group of prey insects (bees, flies and beetles) were photographed was added to the explanatory variables.

Insect group	Explanatory variable	Estimate	Standard error	*z*-value	*P*-value
Bees	(Intercept)	− 18.08250	1.650575	− 10.955	< 0.0001
	Day 2	4.463235	0.132434	33.702	< 0.0001
	Day 3	2.224953	0.138317	16.086	< 0.0001
	Day 4	0.130947	0.236873	0.553	0.58
	Rainfall	− 0.911440	0.076494	− 11.915	< 0.0001
	Wind	− 0.439570	0.085296	− 5.153	< 0.0001
	Temperature	1.733638	0.115517	15.008	< 0.0001
	Temperature^2^	− 0.040340	0.002087	− 19.329	< 0.0001
Flies	(Intercept)	− 54.170	1262	− 0.043	0.966
	Day 2	21.540	1262	0.017	0.986
	Day 3	19.860	1262	0.016	0.987
	Day 4	20.480	1262	0.016	0.987
	Rainfall	− 20.360	1888	− 0.011	0.991
	Wind	− 1.800	0.199	− 9.040	< 0.0001
	Temperature	2.593	0.142	18.265	< 0.0001
	Temperature^2^	− 0.050	0.002	− 19.935	< 0.0001
Beetles	(Intercept)	− 134.400	1200	− 0.112	0.911
	Day 2	20.920	1200	0.017	0.986
	Day 3	22.320	1200	0.019	0.985
	Day 4	19.860	1200	0.017	0.987
	Rainfall	− 18.810	32,170	− 0.001	1
	Wind	0.704	0.070	10.058	< 0.0001
	Temperature	7.576	0.187	40.529	< 0.0001
	Temperature^2^	− 0.129	0.003	− 40.889	< 0.0001
Wasps	(Intercept)	− 193.700	271	− 0.716	0.47404
	Day 2	23.240	270	0.086	0.93140
	Day 3	21.650	270	0.080	0.93606
	Day 4	− 4.270	737	− 0.006	0.99537
	Rainfall	− 25.720	1024	− 0.025	0.97996
	Wind	− 0.988	0.388	− 2.548	0.01083
	Number of bees	0.003	0.002	1.700	0.08915
	Number of flies	0.003	0.003	0.935	0.34994
	Number of beetles	0.001	0.001	2.553	0.01069
	Temperature	11.930	3.769	3.164	0.00156
	Temperature^2^	− 0.212	0.066	− 3.214	0.00131

### Insect groups and time periods with strong relationships with seed set

**Figure 3:**
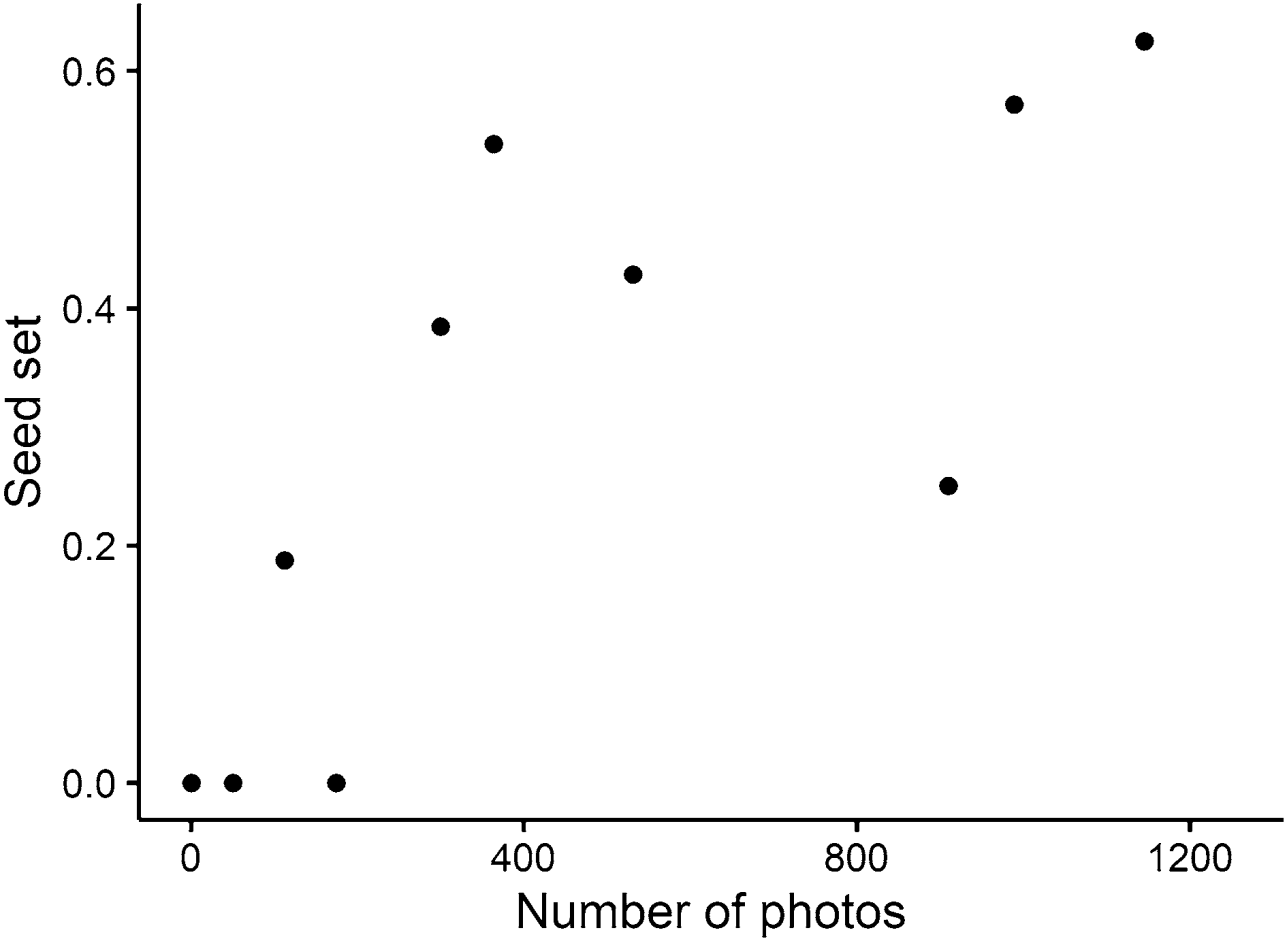
Relationship between the number of photos of flower-visiting bees on Day 2 and seed set (number of mature seeds divided by ovule number in a flower).

**Figure 4:**
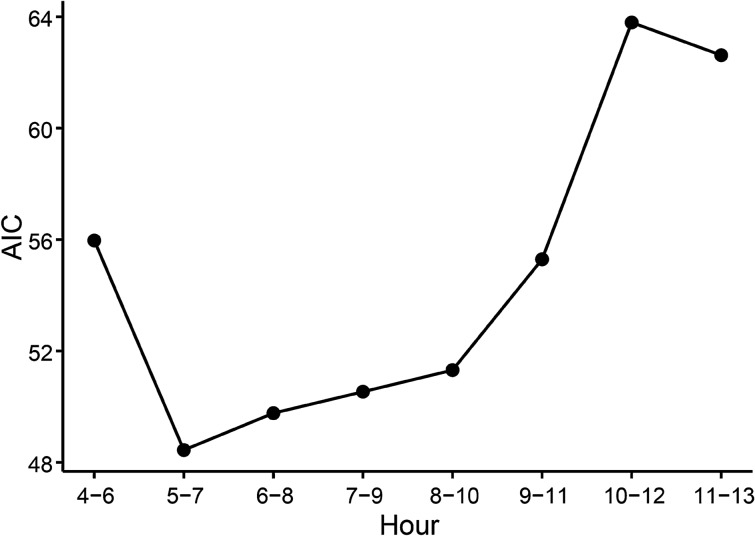
Change in 3-h AIC with shifts in the time period by 1 h in logistic regression analysis of the effect of the number of bees photographed on Day 2 on seed set. The pollinator group and Day were selected by former analysis that contributed to seed set of lotus flowers in this study. Lower value of AIC indicates more effective time period of visitation to seed set.

The total number of photos taken of flower-visiting insects (bees, flies, beetles, moths and unknown) on Day 2 had a significant positive effect on seed set of the 10 flowers from which intact fruits were harvested (Table 2). On Day 2, the relationship between the number of photos taken and the seed set in each insect group, excluding moths, showed a significant positive effect of bees (estimate = 0.0016, *z* = 2.570, *P* = 0.0102, Fig. 3), but no effect of flies (estimate = 0.0006, *z* = 0.808, *P* = 0.4189) or beetles (estimate = 0.0005, *z* = 0.832, *P* = 0.4055). AIC was the lowest, at 48.44, at 05:00–07:59 (Fig. 4), suggesting that bee visits during this period on Day 2 are particularly effective for seed set. The number of bee photos taken between 05:00 and 07:59 on Day 2 had a significant effect on seed set, even when floral traits and flowering date were included in the analysis (Table 3).

**Table 2:** Results of logistic regression analysis examining the effects of the number of photos of potential pollinators taken at floral reproductive organs of lotus flowers during each Day of flowering on seed set. The number of photos of all flower-visiting pollinator insects on each Day was summed up. The number of mature seeds was assumed to follow a binomial distribution, with the number of ovules in the flower as the upper limit. Before the analysis, it was confirmed that there was no multicollinearity among the explanatory variables.

Explanatory variable	Estimate	Standard error	*z-*value	*P-*value
(Intercept)	− 1.4053645	0.390098	− 3.603	0.000315
Day 1	− 0.0049109	0.025272	− 0.194	0.845922
Day 2	0.0011096	0.000373	2.978	0.002903
Day 3	− 0.0013500	0.000630	− 2.143	0.032152
Day 4	0.0018241	0.002438	0.748	0.454398

**Table 3:** Results of logistic regression analysis examining factors affecting seed set of lotus flowers. The number of photos taken of bees during 05:00–07:59 (the time period when the AIC value was lowest), petal type (single or double), number of ovules (an index of flower size) and Julian date (number of days from 1 April 2018 to day 1) were used as explanatory variables. The number of mature seeds per flower was assumed to follow a binomial distribution with the number of ovules as the upper limit.

Explanatory variable	Estimate	Standard error	*z-*value	*P-*value
(Intercept)	− 117.5000	59.7300	− 1.968	0.04912
Single petal type	− 1.0820	0.7038	− 1.537	0.12431
Flower size	0.0918	0.0647	1.420	0.15553
Bees’ visitation during 05:00–07:59 on Day 2	0.0029	0.0010	2.836	0.00457
Julian date	1.8540	0.9350	1.983	0.04734
Julian date^2^	− 0.0074	0.0037	− 2.018	0.04361

### Relationship between predators and pollinator insects on flowers

Two species of wasps were photographed on and around 5 flowers: *Vespa simillima xanthoptera* was found in 1 series of 2 photos on cultivar ‘OGRN’, and *Vespa analis insularis* was found in 1 photo on ‘KOCH’, 1 on ‘SKHN’, 2 on ‘BTNM’, and 59 series of 75 photos on ‘TNSH’. The number of photos was not influenced by the Day but tended to be higher during hours when there was more prey to hunt on the flower—especially beetles (Table 1). No visitation was observed during rainfall. As in pollinator insects, significant effects of temperature and its square were detected (Table 1). ‘TNSH’, which wasps visited very often, had relatively low bee visitation (180 times on ‘TNSH’ vs 512 times on average on other cultivars) and set no seeds.

We observed 8 wasps hunting, 10 searching, 42 waiting, 16 flying, and 5 perching (Supplementary Fig. S3). We recorded 3 hunting attempts to *G. jucunda*, 2 to *P. japonica*, 1 to *S. obsoleta* and 2 to an unknown species, which resulted in 1 success each for *S. obsoleta* and the unknown.

**Figure 5:**
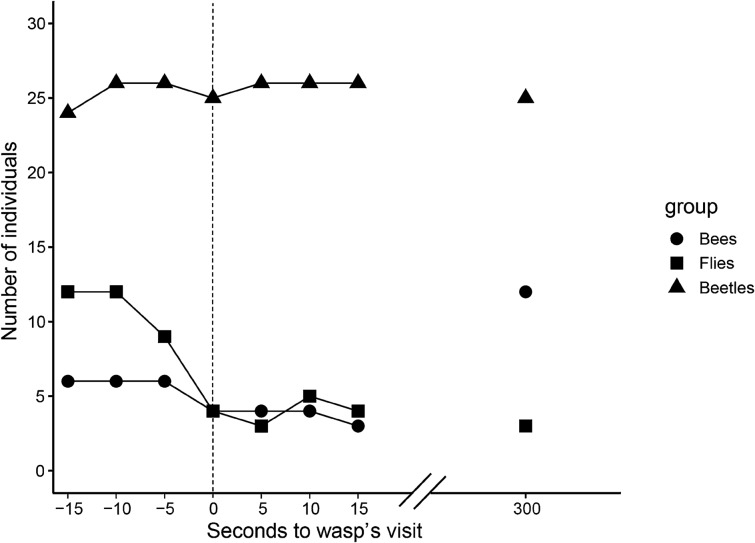
Time course of change in the number of flower-visiting insects by taxonomic group before and after the arrival of wasps. Dashed line shows the time of wasps’ arrival to the flower. Data for all flowers examined were summed.

The number of flower-visiting insects tended to decrease gradually from 15 s before to 5 s after the arrival of wasps, but the pattern differed among insect groups (Fig. 5). Flies left flowers the most (8 times) during and immediately after a wasp’s arrival, followed by bees (3 times). The number of beetles was almost unchanged. At 5 min (300 s) after the arrival of wasps, the number of beetles did not change, bee numbers increased to more than before, and the number of flies, which decreased greatly on the arrival of the wasps, did not recover (Fig. 5).

Spiders were found on 3 flowers: 128–236 times a day on Days 1–4 on ‘AIRN’, 3 times on Day 2 on ‘CHRN’ and 467 times on Day 4 on ‘JSTU’. The actual number of flower-visiting spiders was estimated to be very small because all the pictures in a day were taken in sequence. We did not find any flower-visiting insects being caught by spiders in the photos. The flower of ‘AIRN’, on which spiders were observed every day, had very few visitations by pollinator insects (26 times in total over 4 days of flowering) and set no seeds.

## Discussion

Nearly complete photographic observation of the flower-visiting patterns of pollinator insects to lotus flowers during the entire flowering period revealed that bee visits during the early morning hours of 05:00–07:59 on Day 2 contributed strongly to seed set. It is recommended to estimate pollination efficiency or pollinator effectiveness^49^ by using two or more indices connected to any of flower visitation, pollen deposition or seed set^50^. If one wants to connect pollinator visitation to plant reproductive success, it must be the most reliable to check all the flower-visiting events. However, most classical pollination studies observed floral visitors in a limited time period to estimate effective pollinator candidates^51^. Even the researchers who payed attention to the change in patterns of insect activity or flower visitation over time conducted the observations not continuously all day long and during all flowering period but several times a day with several tens of minutes to few hour interval^17,18,21,23^. To our knowledge, the only study that investigated flower visitation pattern by pollinators during all flowering period was that of Edwards et al.^22^. The notable achievement of our study was specifying efficient pollinator taxa by examining not only the pollinator activity but also the following reproductive success of the same flower. The design of this study allowed us to detect near-complete flower visitation events during flowering, to clarify differences in flower visitation patterns among taxa, and to narrow down which groups of insects and the times when they visited flowers were linked directly to seed set. While not ‘complete’, as it is possible that we may have missed brief flower visits of less than 5 s, mainly by bee species, the main results will remain the same. Additional pollen deposition experiments, such as counting the number of pollen grains attached to the body surfaces of visitor species or the stigmatic pollen deposition per single visit^11^, can offer supporting evidence of effective pollination by bees.

Among the bees that were most effective in seed set of lotus flowers here, the honeybee has been best studied for its ecology and behaviour, as it is one of the most important pollinators of crops^52,53^. It generally spends a long time outside the hive foraging—from sunrise to sunset^54^—but here, its visitations to lotus flowers were concentrated in the early morning, as reported of the nectarless Mexican cantaloupe^55^. It is said that bees have a fixed time of the day to visit the flowers of each plant species (‘time-memory’^56^). On the other hand, beetle species were found on flowers until evening. These varied patterns of visitation indicate that, to understand the dynamics of flower–pollinator mutualistic interactions, we need to observe all visitors to a flower during its entire blooming period^22^, especially in systems involving multiple pollinator species with different visiting patterns.

The photos also revealed changes in flower visitation patterns with changes in rain, wind and temperature. Precipitation reduced the visitation frequency by all visitors. Although rain had a significant negative effect only on bees, each cultivar had a different flowering period (so some cultivars did not experience rainy weather at all during flowering), and there was large amount of zero data overall. It was clear that the visitation frequencies of all insects decreased under strong winds and under low and high temperatures. Pollinator studies in the field are usually conducted only under optimal weather conditions, and there are few examples of the measurement and detection of weather effects on flower-visiting behaviour^57,58^. The pattern that we detected was reasonable in the context of insect morphology and behaviour. It will be necessary to explicitly test whether this pattern affects plant reproductive success.

Consecutive time-lapse photography revealed not only the mutualistic relationship between plants and pollinators, but also more complex interactions among the plants, the pollinators (as pollen eaters), and their predators on the flowers. The decline of pollinators caused by the approach of wasps suggests that pollinators may initially flee. These rare but important events that can influence the plant reproductive success must be difficult to be detected in short-time observations. Differences in the responses of the pollinator groups to the approach of wasps were explained by the differences in visitation frequency and visitation time. Honeybees tended to stay for only 5–10 s and leave, regardless of the arrival or absence of wasps, and their high visitation frequency could have masked any decline. Halictid bees, which stayed longer than the honeybees, reacted immediately to attack by wasps and left the flower, and only after the wasps had left, they did return. Visitation by flies, at low frequency but long duration, did not recover quickly after wasps attacked. Beetles were the most sluggish and were sometimes attacked by wasps, but they did not appear to be successfully hunted and remained on the flower. Collection of such data may help to clarify the interactions among three or more species in a food web at the behavioural level. Despite being documented as major prey of *V. simillima xanthoptera* and *V. analis insularis* in Japan^59^, bees—the most frequently observed insects in this study—were not attacked by wasps. Hymenopteran insects are known to be more likely than dipterans to avoid flowers with predators or evidence of predators^60,61^, and *A. cerana* avoids nectar sources when wasps are present nearby^20^. The result of few bee visitations to, and fruiting failure of, the ‘TNSH’ flower may be an example of a negative effect of predators on the reproductive success of plants by reducing the frequency of visitation by effective pollinators. The presence of spiders may also reduce the success of pollination of lotus flowers. The low visitation frequency by pollinators and fruiting failure of the ‘AIRN’ flower may be the result of the recognition of spiders and the subsequent avoidance of the flower by pollinator species, as has been confirmed experimentally with sit-and-wait crab spiders^61^. Further investigations are needed to test these possibilities.

We identified effective pollinators of cultivated lotus. Our method could be applied effectively to crops grown under controlled environments. Populations of honey bees^62–65^ and other wild bees^66,67^ have declined globally in recent years. The importance of maintaining an abundance of wild insect fauna that can serve as crop pollinators is increasingly recognized^5,68,69^, but few studies have examined the effectiveness of wild pollinators at pollinating and fruiting crops^70,71^. Clarification of multiple individual pollination systems could support the worldwide conservation of ecosystem services.

Although not accomplished species identification from the photos, our system classified 96% of observed pollinator individuals into the orders, so it will work sufficiently for studies of natural history of pollination system or pollination syndrome. It is especially suitable for the combination of large, flatly-open diurnal flowers and pollinator insects with high flying ability. However, it may not be practical to apply to plants that flower at night. Nocturnal pollinators play an important role in the reproductive success of nocturnal flowering plants^72^, but camera lighting at night may affect their pollinators’ behaviour^73^. Night photography techniques that capture clear images for species-level identification without interfering with pollinator activity must be developed. Another problem with the application of our system to other plant species is that pollinator detection rates may be dropped in flowers with complex spatial structure^74^ or for mass tiny insect pollinators. For those species, multiple cameras from different angles may be required for each target flower^33^. Methods for pollinator species identification must also be improved. Here, insects were detected and identified by eye in a large number of photos. These tasks could be automated through technological developments in object detection, such as deep learning^29,75,76^.

Our understanding of pollination interactions, hitherto based mainly on direct observation for a limited time span, could be dramatically improved by using weather-proof automatic cameras, as shown here. We revealed the effective pollinator taxa and the timing of their visitation to the reproductive success of lotus flowers under the complex biological interactions and fluctuating abiotic factors. This study shows the availability of periodical photographing method in clarifying the plant-pollinator interaction and its consequence (reproductive success of the plant) more in detail than traditional direct observations in pollination studies. Such a method not only would enable us to directly analyze the consequences of insects’ visits on seed set, but also has the potential to clarify the food web relationships among plants (flowers and fruits), flower-visiting insects and predatory arthropods at the behavioural level. Consecutive photography system with high-resolution SLR camera will identify almost all individuals to species level including tiny insects and help answer the species-focused questions, such as how many pollinator species are there or whether a concerned pollinator species visit a target plants. Then the challenge will be to build a monitoring system that can collect higher-resolution time-lapse images and an efficient algorithm to generate large amounts of training data that cover most of the visiting insect species in natural environments^77^.

## Supplementary Information

Supplementary Information.

## Data Availability

The datasets generated or analysed during this study are available from the corresponding author on reasonable request.
