# Supplementary material for: Periodically taken photographs reveal the effect of pollinator insects on seed set in lotus flowers
Source: Sci Rep. 2022 Jul 11;12:11051. doi: 10.1038/s41598-022-15090-0 (PMC9273618; doi:10.1038/s41598-022-15090-0)
Supplement: Supplementary file 1 — Supplementary Information. [file 41598_2022_15090_MOESM1_ESM.pdf]

Supplemetary Tables and Figures

Periodically taken photographs reveal the effect of pollinator insects on seed set in lotus flowers

Mihoko Nagai, Yohei Higuchi, Yusei Ishikawa, Wei Guo, Tokihiro Fukatsu, Yuki G. Baba, and Mayura B. Takada

Supplementary Table S1. Name, flowering date, petal type, and numbers of petals, pistils, stamens and mature seeds of each focal flower. The numbers of stamen are given as approximates.
*Not counted because the fruit receptacles were lost; see Materials and methods.

Cultivar name	Flowering date	Petal type	#petal	#pistil	#stamen	#seed
'Unknown (UNKN)'	2018/6/30	single	15	4	50	NA*
'Chirinoakebono (CHRN)'	2018/7/11	single	12	23	250	NA*
'Betonamumomo (BTNM)'	2018/7/13	double	50	32	300	20
'Sokuhiren (SKHN)'	2018/7/20	single	19	14	150	8
'Maihiren (MHRN)'	2018/7/25	single	11	13	100	5
'Airairen (AIRN)	2018/8/01	double	40	4	10	0
'Oguranokagayaki (OGRN)'	2018/8/02	single	13	16	80	3
'Kououchou (KOCH)'	2018/8/12	single	15	14	60	6
'Heisanfuyou (HSFY)'	2018/8/18	double	50	5	40	0
'Juseitou (JSTU)'	2018/8/19	double	40	13	80	7
'Zuikouren (ZKRN)'	2018/8/22	single	12	16	100	4
'Tenshou (TNSH)'	2018/8/24	double	50	16	100	0

Supplementary Table S2. Number of photos taken of flower-visiting arthropods each day after flowering.

Group	Subgroup	Day 1	Day 2	Day 3	Day 4	Total
Potential pollinators at floral reproductive organs						
Hymenoptera	*Apis* sp.	17	3,374	487	5	3,883
	Xylocopa appendiculata circumvolans	0	36	14	0	50
	Halictidae spp.	41	1,766	56	21	1,884
Hymenoptera subtotal		58	5,176	557	26	5,817
Diptera	*Stomorhina obsoleta*	0	1,545	403	354	2,302
	Phytomia zonata	0	14	0	0	14
	other Syrphidae	0	20	0	0	20
Diptera subtotal		0	1,579	403	354	2,336
Coleoptera	*Gametis jucunda*	0	13	1,198	99	1,310
	Popillia japonica	0	1,168	750	0	1,918
	not identified	0	244	290	1	535
Coleoptera subtotal		0	1,425	2,238	100	3,763
Lepidoptera		0	4	1	128	133
Unknown		4	496	176	106	782
Potential pollinators subtotal		62	8,680	3,375	714	12,831
Predators on flowers						
Wasps	*Vespa analis insularis*	0	56	23	0	79
	Vespa simillima xanthoptera	0	2	0	0	2
Wasps subtotal		0	58	23	0	81
Spiders		139	151	236	595	1,121
All Total		201	8,889	3,634	1,309	14,033

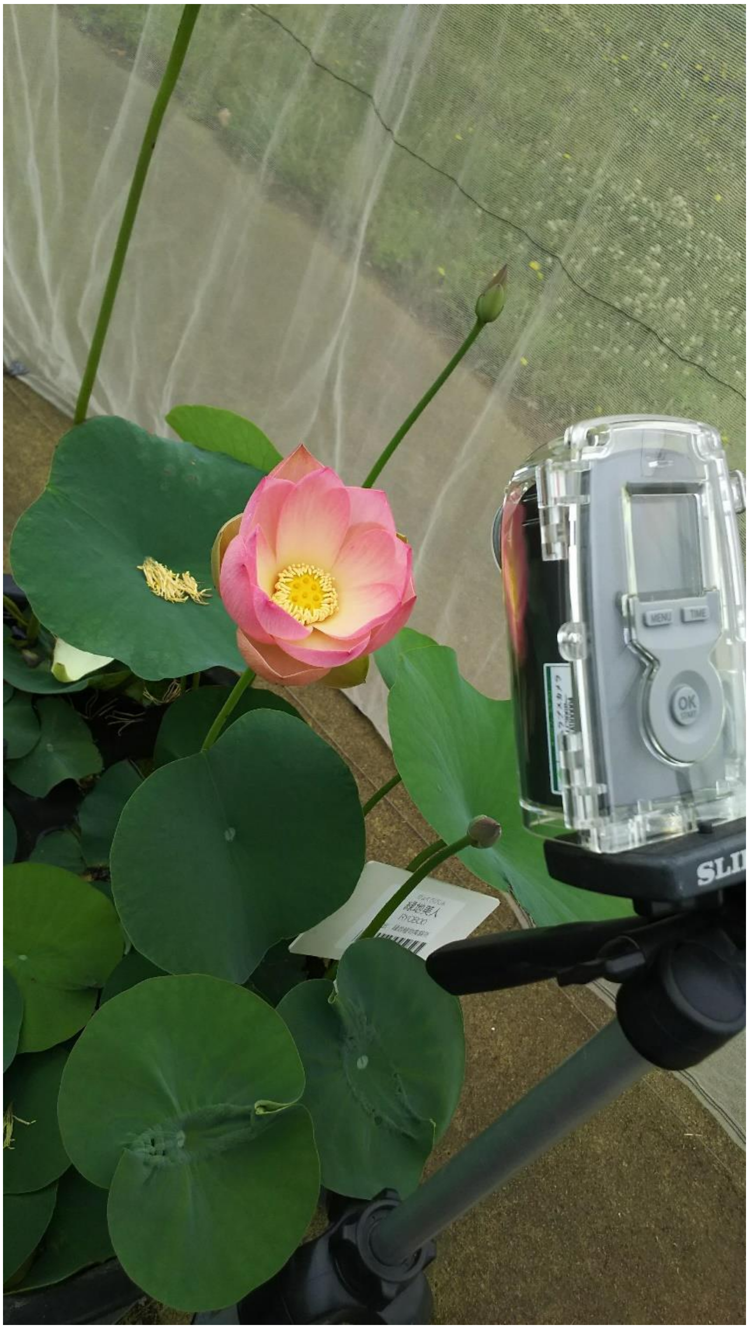

Supplementary Figure S1.

Photographic system .used in this study. The camera (TLC200 Pro, Brinno) was held inside a semitransparent plastic weather-resistant housing and fixed on a tripod. Four AA butteries and a 32-GB SD card were set inside the camera. The camera lens was then fixed facing the opening of the flower. Photos were taken periodically every five seconds from dawn to dusk during flowering period.

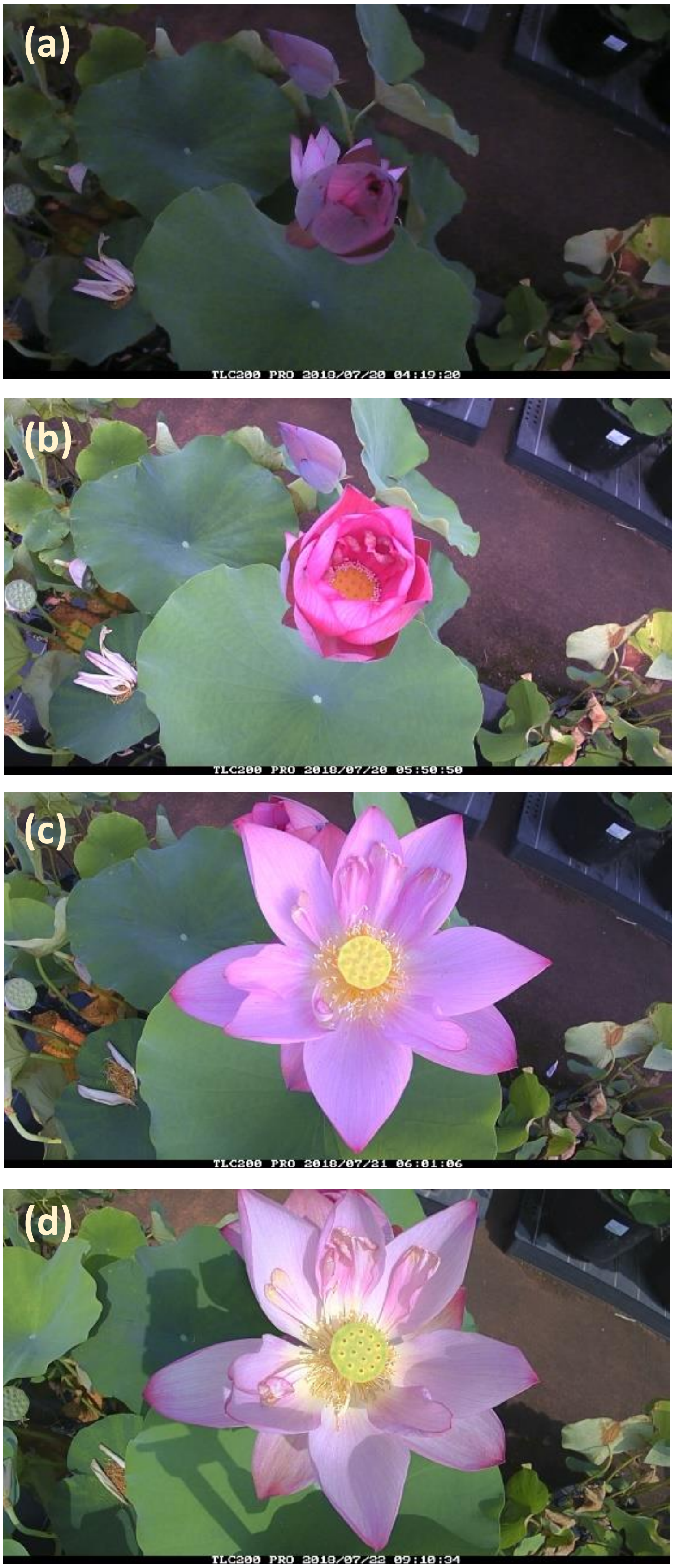

Supplementary Figure S2. Flowering phenology of cultivar SKHN. **(a)** Flower opening on Day 1, **(b)** maximum open on Day 1, **(c)** maximum open on Day 2, **(d)** maximum open on Day 3. On Day 3, the stigma surface browned, suggesting receptivity was lost.

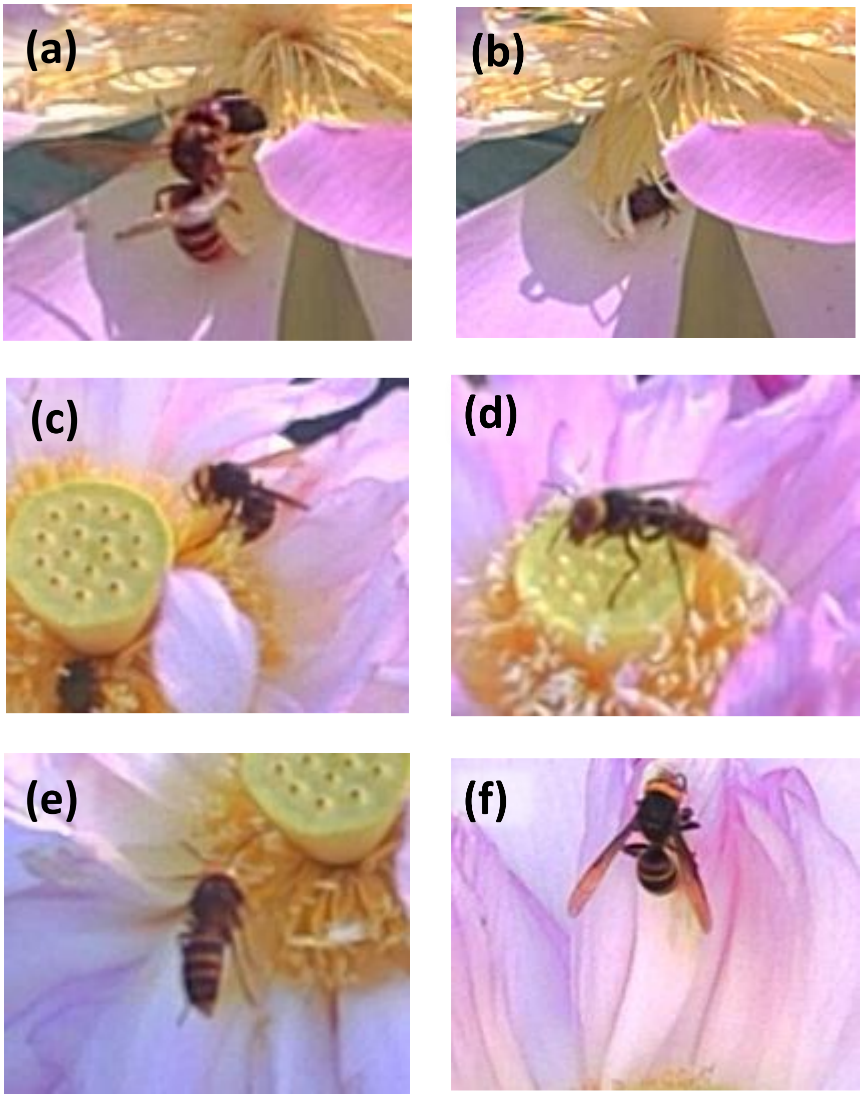

Supplementary Figure S3. Photos with a wasp that was **(a)** hunting, **(c)** searching, **(d)** waiting, **(e)** flying, and **(f)** perching. **(b)** If the same kind of insect was still there after attack by a wasp, the hunt was judged as “failed” (5 s after **a**).
